# *%diag_test*: a generic SAS macro for evaluating diagnostic accuracy measures for multiple diagnostic tests

**DOI:** 10.1186/s12911-024-02808-5

**Published:** 2025-01-13

**Authors:** Jacques K. Muthusi, Peter W. Young, Frankline O. Mboya, Samuel M. Mwalili

**Affiliations:** 1 Division of Global HIV and Tuberculosis, Global Health Centre, U.S. Centres for Disease Control and Prevention, P.O. Box 606 – 00621, Nairobi, Kenya; 2 Division of Global HIV and Tuberculosis, Global Health Centre, U.S. Centres for Disease Control and Prevention, Maputo, Mozambique; 3 Institute of Mathematical Sciences Centre for Health Analytics and Modelling (CHaM), Strathmore University, Nairobi, Kenya

**Keywords:** SAS macro, Disease prevalence, Diagnostic accuracy measures, Reproducible research, Machine learning classification

## Abstract

**Background:**

Measures of diagnostic test accuracy provide evidence of how well a test correctly identifies or rules-out disease. Commonly used diagnostic accuracy measures (DAMs) include sensitivity and specificity, predictive values, likelihood ratios, area under the receiver operator characteristic curve (AUROC), area under precision-recall curves (AUPRC), diagnostic effectiveness (accuracy), disease prevalence, and diagnostic odds ratio (DOR) etc. Most available analysis tools perform accuracy testing for a single diagnostic test using summarized data. We developed a SAS macro for evaluating multiple diagnostic tests using individual-level data that creates a 2 × 2 summary table, AUROC and AUPRC as part of output.

**Methods:**

The SAS macro presented here is automated to reduce analysis time and transcription errors. It is simple to use as the user only needs to specify the input dataset, “standard” and “test” variables and threshold values. It creates a publication-quality output in Microsoft Word and Excel showing more than 15 different accuracy measures together with overlaid AUROC and AUPRC graphics to help the researcher in making decisions to adopt or reject diagnostic tests. Further, it provides for additional variance estimation methods other than the normal distribution approximation.

**Results:**

We tested the macro for quality control purposes by reproducing results from published work on evaluation of multiple types of dried blood spots (DBS) as an alternative for human immunodeficiency virus (HIV) viral load (VL) monitoring in resource-limited settings compared to plasma, the gold-standard. Plasma viral load reagents are costly, and blood must be prepared in a reference laboratory setting by a qualified technician. On the other hand, DBS are easy to prepare without these restrictions. This study evaluated the suitability of DBS from venous, microcapillary and direct spotting DBS, hence multiple diagnostic tests which were compared to plasma specimen. We also used the macro to reproduce results of published work on evaluating performance of multiple classification machine learning algorithms for predicting coronary artery disease.

**Conclusion:**

The SAS macro presented here is a powerful analytic tool for analyzing data from multiple diagnostic tests. The SAS programmer can modify the source code to include other diagnostic measures and variance estimation methods. By automating analysis, the macro adds value by saving analysis time, reducing transcription errors, and producing publication-quality outputs.

**Supplementary Information:**

The online version contains supplementary material available at 10.1186/s12911-024-02808-5.

## Background

Disease-causing organisms in humans include viruses, bacteria, fungi, protozoa, and helminths (worms). For instance, tuberculosis (TB) is caused by the *Mycobacterium tuberculosis* bacterium whereas acquired immune deficiency syndrome (AIDS) is caused by the human immunodeficiency virus (HIV). Other diseases like hypertension, diabetes or obesity can be caused by unhealthy lifestyle choices, genetic predisposition, or a combination of factors [1–3]. To diagnose medical conditions, a clinician may obtain a specimen (e.g., sample of blood, urine, stool, sputum, etc.) for laboratory testing. Many laboratory devices are calibrated to give test results on a continuous scale (e.g., count of bacteria or viruses in the specimen or level of blood pressure), while others provide qualitative responses (e.g., present or absent). In the case of continuous measurements, reference ranges are provided to the clinician. Values of test results outside the reference range are considered abnormal and provide evidence for diagnosis of the disease under investigation [4]. For instance, a patient is considered hypertensive if values of systolic and diastolic measurements are higher than 140 and 90 mm Hg respectively [5, 6]. In HIV programming, an individual’s viral load is considered suppressed if their viral load test result yield viral copies less than or equal to 1,000 copies/mL [7].

Measures of diagnostic accuracy are used to demonstrate the ability of a diagnostic test (or procedure or device) in correctly identifying the presence and absence of a disease condition in comparison to a reference standard [8]. Many such measures start by categorizing the continuous values from the test into binary (e.g., presence or absence of disease) or multicategory classes (e.g., hypotension, normal blood pressure or hypertension). Some commonly used DAMs include sensitivity and specificity, predictive values, misclassification likelihood ratios, diagnostic effectiveness (accuracy) and diagnostic odds ratio (DOR) [9, 10]. Other measures include Kappa coefficient [11], Youden’s index [12], F-score [13], receiver operating characteristic (ROC) curve [14] and precision-recall (PR) curve [15].

New diagnostic methods or tests may be proposed to improve performance and reduce the cost, complexity, invasiveness, or turnaround time of current practice. When doing so, it is important to characterize how well the new test performs compared to current practice. Measures of diagnostic accuracy compare the result obtained on a new diagnostic test with a ‘gold-standard’ or reference diagnostic test that is thought to be more accurate than the test under evaluation. In practice, even the reference standard may not be perfectly accurate as many conditions cannot be measured with complete certainty [16].

DAMs for a single test can be computed using most available statistical software including Statistical Analysis System (SAS), STATA^®^, and R. For example in SAS, one can use the procedures PROC FREQ or PROC SURVEYFREQ [17] to compute DAMs. In Stata, one can use **diagt** [18] or **roctabi** [19] commands. In R software, several meta packages have been developed including “***metaprop***” and “***metabin***” for sensitivity, specificity, and diagnostic odds ratio [20]. There are also several online calculators that have been developed to perform diagnostic accuracy testing. Though they provide estimates for most-used diagnostic measures, these tools suffer challenges such as lack of flexibility to use patient-level data, inability to evaluate multiple tests iteratively, and inaccurate confidence interval estimation. For instance, MedCalc [21], Schwartz [22] and Chatzimichail [23], have developed online calculators whose purpose is exploratory and educational that use the normal distribution approximation which yield intervals with poor coverage when used with small sample sizes [24, 25]. The online calculators further require the analyst to aggregate the data first, can only evaluate one diagnostic test at a time, and do not allow the user to modify the source code.

To overcome these challenges, we developed a generic SAS macro for computing DAMs on subject-level data. The macro can be applied in different settings such as the laboratory to evaluate performance of multiple diagnostic tests or in machine learning to evaluate performance of different classification algorithms. It provides the user with several methods of computing confidence intervals for the DAMs point estimates. It is further helpful in situations where no gold standard or reference method exists.

## Methods

### Diagnostic accuracy measures

Commonly used qualitative DAMs are briefly described here. All the measures presented here assume the disease status has been independently ascertained through a reference test. Sensitivity (true positive rate (TPR) or recall) and specificity (true negative rate (TNR)) are the probability of a correct test result in subjects with and without a condition, respectively [26]. Predictive values are the probability of correctly identifying a subject’s condition given the test result and can either be positive (precision) or negative. False omission rate (FOR) is the complement of negative predictive value (NPV) [27, 28]. Likelihood ratios are used for assessing the value of performing a diagnostic test and can either be for positive or negative test result [29, 30]. Upward misclassification (also referred to as the false positive rate (FPR)) is the proportion of all negatives that still yield positive test outcomes and is usually equal to the significance level (Type I error, *α*). Downward misclassification (also referred to as the false negative rate (FNR)) is the proportion of positives which yield negative test outcomes with the test and is the equivalent of Type II error, *β*, in statistical hypothesis testing [31]. False discovery rate (FDR) is the expected ratio of the number of false positive classifications (false discoveries) to the total number of positive classifications (rejections of the null) [32, 33]. A DAM expresses how well a diagnostic test under evaluation correctly identifies or rules out disease by comparison with a reference standard of the “true” disease status (both true positives and true negatives) among the total number of subjects examined. This is usually affected by the disease prevalence. With the same sensitivity and specificity, diagnostic accuracy of a particular test increases as the disease prevalence decreases [34]. Disease prevalence refers to the proportion of a population found to be affected by a disease at a specific time [35]. Diagnostic odds ratio (DOR) is referred to as the effectiveness of a diagnostic test and is defined as the ratio of the odds of the test being positive if the subject has a disease relative to the odds of the test being positive if the subject does not have the disease. DOR depends on both the sensitivity and specificity of a test. For instance, a test with high specificity and sensitivity (or with a low rate of false positives and false negatives) has high DOR. With the same sensitivity of the test, DOR increases with the increase of the test specificity [36]. F-score (or F-measure) is a measure of a test’s accuracy and is calculated from the precision (positive predictive value) and recall (sensitivity) of the test [13]. Youden’s Index provides another way of summarizing the performance of a diagnostic test. It takes values in the ranges from 0 to 1 (inclusive). A value of 0 indicates same proportion of positive results for groups with and without the disease (diagnostic test is less useful) whereas a value of 1 indicates that there are no false positives or false negatives (diagnostic test is perfect). Youden’s Index is affected by spectrum of the disease rather than disease prevalence [12]. Cohen’s kappa coefficient (κ) is used to measure the agreement between two raters who each classify N items into C mutually exclusive categories [11]. Finally, the ROC curves is a graphical presentation that illustrates the diagnostic ability of a binary classifier system as its discrimination threshold is varied [37] and the PR curve graphs precision-recall trade-offs at different probability thresholds [38].

In medical epidemiology, when a diagnostic test produces a continuous measure, there is usually a cutoff value indicating whether a subject should be classified as having the disease or condition (above/below the cutoff) or not (below/above the cutoff). When evaluating the performance of a diagnostic test, we are comparing to a gold standard (or reference method) which is believed to indicate with greater accuracy whether the same subject has the disease or not. The combination of the diagnostic test and the gold standard divide the population of examined subjects into four subgroups, generally displayed in a 2 × 2 contingency table as show in Table 1 [9].

**Table 1:** Sample 2 × 2 contingency table used to report cross-classification of subjects

	Reference test		
Diagnostic test	Subjects with disease	Subjects without disease	Total
Positive	TP = *a*	FP = *b*	TP + FP = *n1*
Negative	FN = *c*	TN = *d*	FN + TN = *n2*
Total	TP + FN = *m1*	FP + TN = *m2*	Total = *n*

Here, true positive (TP) refers to subjects with the disease with the value of a variable of interest above/below the cutoff while false positive (FP) refer to subjects without the disease with the value of a variable of interest above/below the cutoff. On the other hand, true negative (TN) refers to subjects without the disease with the value of a variable of interest below/above the cutoff whereas false negative (FN) refers to subjects with the disease with the value of a variable of interest below/above the cutoff. The SAS macro uses the quantities $\:a,b,c,d,\:n1,\:n2,\:m1,\:m2\:\text{a}\text{n}\text{d}\:n$ for ease of coding. Formulas for computation for each measure of diagnostic accuracy are presented in Table 2.

**Table 2:** Formulas for computation of qualitative DAMs

Measure	Formula
Sensitivity or True Positive Rate (TPR)	TP / (TP + FN)
Specificity or True Negative Rate (TNR)	TN / (TN + FP)
Positive Predictive Values (PPV)	TP / (TP + FP)
Negative Predictive Value (NPV)	TN / (TN + FN)
Upward Misclassification or False Positive Rate (FPR) or Type I error	FP / (FP + TN) = 1 – Specificity
Downward Misclassification or False Negative Rate (FNR) or Type II Error	FN / (TP + FN) = 1 – Sensitivity
False Omission Rate (FOR)	FN / (FN + TN) = 1 - NPV
False Discovery Rate (FDR)	FP / (TP + FP) = 1 - PPV
Positive Likelihood Ratio (LR+)	Sensitivity / (1 – Specificity)
Negative Likelihood Ratio (LR–)	(1 – Sensitivity) / Specificity
Diagnostic Accuracy	(TP + TN) / (TP + FP + TN + FN)
Disease Prevalence	(TP + FN) / (TP + FP + TN + FN)
Diagnostic Odds Ratio (DOR)	LR+ / LR– = (TP / FN) / (FP / TN)
Youden’s Index	Sensitivity + Specificity − 1
Kappa coefficient	(po – pe) / (1 – pe) where; po = (TP + TN) / (TP + FP + TN + FN) pe = ((TP + FN) * (TP + FP) + (FP + TN) * (FN + TN)) / (TP + FP + TN + FN) **2
F-score	2 *(PPV * Sensitivity) / (PPV + Sensitivity) = 2 TP / (2 TP + FP + FN)

### The *%diag_test* SAS macro

**Fig. 1:**
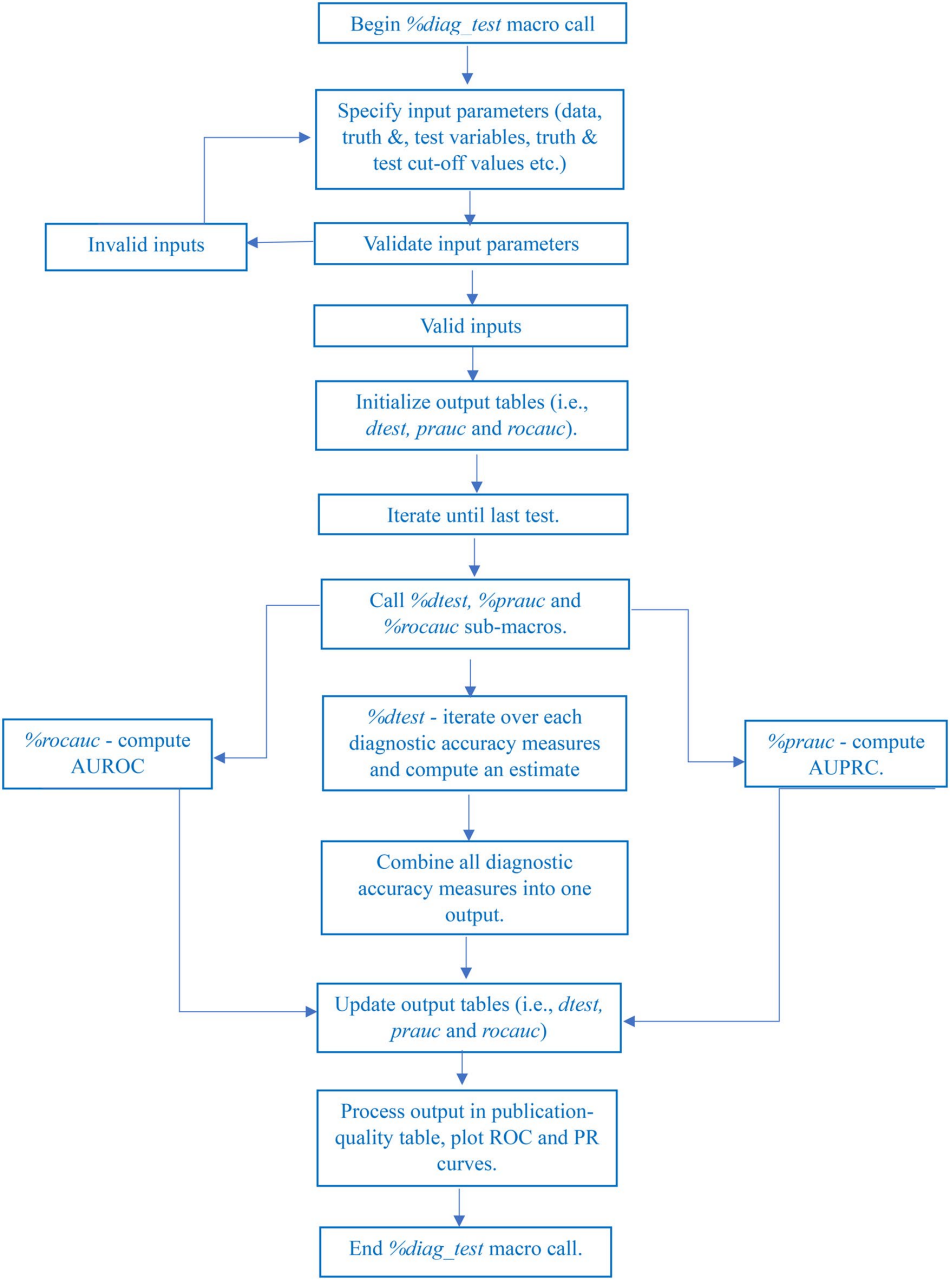
Flowchart showing how *%diag_test* SAS macro works

The SAS macro, *%diag_test*, was written in SAS software version 9.4, copyright © 2022, SAS Institute Inc. with full declaration that SAS and all other SAS Institute Inc. product or service names are registered trademarks or trademarks of SAS Institute Inc., Cary, NC, USA. The macro computes a point estimate for each measure of diagnostic accuracy using the formulae provided in Table 2 and corresponding confidence limits (using several specified methods). It consists of three sub-macros. The first sub-macro *%dtest* computes estimates for each diagnostic test then combines analysis for all of them into one output table. The second sub-macro *%prauc* computes AUPRC and graphs the corresponding PR curve. The third sub-macro *%rocauc* computes AUROC and graphs the corresponding ROC curve. The output is then processed using the PROC TEMPLATE and PROC REPORT procedures and the output delivery system (ODS) to create a publication-quality table. Figure 1 shows a flowchart demonstrating how the SAS macro program works.

The macro enforces validation checks on input parameters (i.e., ensures the user has specified the analysis dataset, reference, and test variables, and corresponding cutoff values) and tests for logical errors for the user to address. The analyst must specify input variables described in Table 3 unless stated “optional” in which case the macro uses default values. For qualitative measures with such values as “Yes/No” or “Present/Absent”, the analyst should recode them into “1/0” format and use a cutoff value of 0.5 to execute the macro. The macro has been developed to run on Microsoft windows platform and might require adjustments to run on other operating systems.

**Table 3:** Input parameters for *%diag_test* macro

parameter	description
data	name of input dataset
truthvar	name of reference/truth/gold standard variable e.g., abbott_plasma_vl
truthcutvalue	cutoff value to use to categorize values of test variable as having disease or not e.g., 1,000 so that if value ≥ 1,000 then presence of disease, otherwise absence of disease
testvarlist	list of diagnostic test variable(s) separated by space e.g., vdbs_vl mdbs_vl ddbs_vl
testcutvalue	cutoff value to use to categorize values of test variable as having disease or not e.g., 1,000 so that if value ≥ 1,000 then presence of disease, otherwise absence of disease
domain	(optional) domain variable for sub-population analysis
domainvalue	(optional) value of domain/sub-population of interest (should be numeric). Required if domain is specified
condition	(optional) any conditional statements to create and or fine-tune the final analysis dataset specified using one IF statement
outputdir	path for directory/folder where output is saved
tablename	short name of output table
tabletitle	title of output table
surveyname	(optional) abbreviation for survey/study to be included in the output
decimalpoints	(optional) desired number of decimal places for each estimated measure (default = 1)
alpha	(optional) desired level of significance (default = 0.05, for 95% confidence intervals)
missvaluelabel	(optional) value label for missing values. If missing data have a format, it should be provided, otherwise macro assumes the default format “.”
varmethod	(optional) method for computing confidence intervals. Included here are: “Normal” (default) for normal distribution approximations, “Wilson” (most desired) for Wilson score and “Exact” for exact binomial approximation
print	variable for displaying/suppressing the output table on the output window which takes the values (NO = suppress output, YES = show output)

### Estimation of confidence intervals

Confidence intervals provide an estimate of the precision of a population variable. Since most measures of qualitative diagnostic accuracy testing follow Bernoulli trials, we construct binomial proportion confidence intervals. Frequently used methods for computing binomial confidence intervals include the normal approximation, Clopper-Pearson interval, and Wilson score methods etc. The normal distribution approximation interval is computed based on a normal distribution approximation of the binomially-distributed observation using the central limit theorem as shown below [39].

1 $$\:\widehat{p}\pm\:{z}_{1-\frac{\alpha\:}{2}}\left(\sqrt{\frac{\widehat{p}\:(1-\widehat{p})}{n}}\right)$$

where, $\:\widehat{p}$ is proportion of interest, $\:n\:$is the sample size, $\:\alpha\:$ is the level of significance (or desired confidence) and $\:{z}_{1-\frac{\alpha\:}{2}}$ is the “Z value” for the desired level of confidence. Though it is easy to implement, this method has been found to be unreliable when the sample size is small, or the success probability is close to 0 or 1. In addition, it can give negative values for the proportion given the assumption of a symmetric distribution [24, 25].

Another early and common method for calculating binomial confidence interval is the Clopper-Pearson interval also known as “Exact” method. This method is based directly on the cumulative probabilities of the binomial distribution rather than an approximation to the binomial distribution and is computed as follows:

2 $$\:B\:\left(\frac{\alpha\:}{2};x;n-x+1\right)<\theta\:<B\left(1-\frac{\alpha\:}{2};x+1;n-x\right)$$

where $\:x$ is the number of successes, $\:n$ is the number of trials, and $\:B(p;\:v,w)$ is the $\:pth$ quantile from a beta distribution with shape parameters $\:v$ and $\:w$ [24]. When $\:x$ is either $\:0$ or $\:n$, closed-form expressions for the interval bounds are available: when $\:x=0$ the interval is $\:\left(\text{0,1}-{\left(\frac{\alpha\:}{2}\right)}^{\frac{1}{n}}\right)\:$and when $\:x=n$ it is $\:\left({\left(\frac{\alpha\:}{2}\right)}^{\frac{1}{n}},1\right)\:$[40]. In cases where the population size is known, the Clopper-Pearson interval becomes less desirable because it may not be the smallest possible.

To overcome challenges with normal approximation and Clopper-Pearson methods, Wallis [39] recommends using the Wilson score method which was developed by Edwin B Wilson [41] because it provides several improvements over the former methods [42, 43]. First, unlike the symmetric normal approximation interval, the Wilson score interval is asymmetric. Second, it does not suffer from problems of overshoot and zero-width intervals that afflict the normal interval. Third, it can be safely used with small samples and skewed observations [24, 25, 39]. Last, the observed coverage probability is consistently closer to the nominal value [44]. The Wilson score interval is computed using Eq. 3:

3 $$\:\frac{\widehat{p}+\:\frac{{z}_{\frac{\alpha\:}{2}}^{2}}{2n}+{z}_{\frac{\alpha\:}{2}}^{2}\sqrt{\frac{\widehat{p}\:(1-\widehat{p})}{n}+\frac{{z}_{\frac{\alpha\:}{2}}^{2}}{4{n}^{2}}}}{1+\:\frac{{z}_{\frac{\alpha\:}{2}}^{2}}{n}}<\theta\:<\frac{\widehat{p}+\:\frac{{z}_{1-\frac{\alpha\:}{2}}^{2}}{2n}+{z}_{1-\frac{\alpha\:}{2}}^{2}\sqrt{\frac{\widehat{p}\:(1-\widehat{p})}{n}+\frac{{z}_{1-\frac{\alpha\:}{2}}^{2}}{4{n}^{2}}}}{1+\:\frac{{z}_{1-\frac{\alpha\:}{2}}^{2}}{n}}$$

Confidence intervals for likelihood ratios and Kappa coefficient were computed using the methods described in [27] and [28] respectively. The SAS macro described here implements the normal distribution approximation (default), Wilson score or Clopper-Pearson exact binomial methods to compute confidence intervals. The SAS programmer can easily modify the code to include other methods of computing binomial confidence intervals.

### Area under the ROC curve

The ROC curve is created by plotting TPR against FPR at various threshold settings [14, 45]. It can also be created by fitting a simple logistic regression model used to study the effect of diagnostic test (continuous scale) on the probability of correctly detecting the disease (gold standard method) using the area under the ROC curve (AUROC) measure which take values ranging from 0 to 1. A value of AUROC = 1 indicates that the diagnostic test can perfectly distinguish between all the ill and healthy subjects correctly, whereas AUROC = 0 indicates the diagnostic test would be predicting all healthy subjects as ill, and all ill as negatives. AUROC values > 0.5 and < 1 indicate there is a high chance that the diagnostic test will distinguish the ill subjects from the healthy ones. AUROC = 0.5 indicate that the diagnostic test is not able to distinguish between ill and healthy subjects properly. This means the diagnostic test is either predicting a random class or constant class for all subjects. Therefore, the higher the AUROC value, the better the diagnostic test’ ability to distinguish between ill and healthy subjects [46].

### Area under the precision-recall curve

Area under the Precision-Recall curve (AUPRC) provides a trade-off between PPV and TPR across various probability threshold and takes the values between 0 and 1 [15, 47]. In most biomedical settings, ill subjects are usually fewer than healthy subjects an indicator of skewed data. In such cases, interest will be on the ill subjects, hence the PR curve becomes more informative than ROC curve. In addition, the AUPRC focuses mainly on the ill subjects (PPV and TPR)) compared to the health ones [38, 48, 49]. The SAS macro includes an output of overlaid ROC and PR curves and corresponding AUC values for the multiple diagnostic tests.

## Results

### Example: application of *%diag_test* macro to evaluate performance of multiple diagnostic laboratory tests

**Fig. 2:**
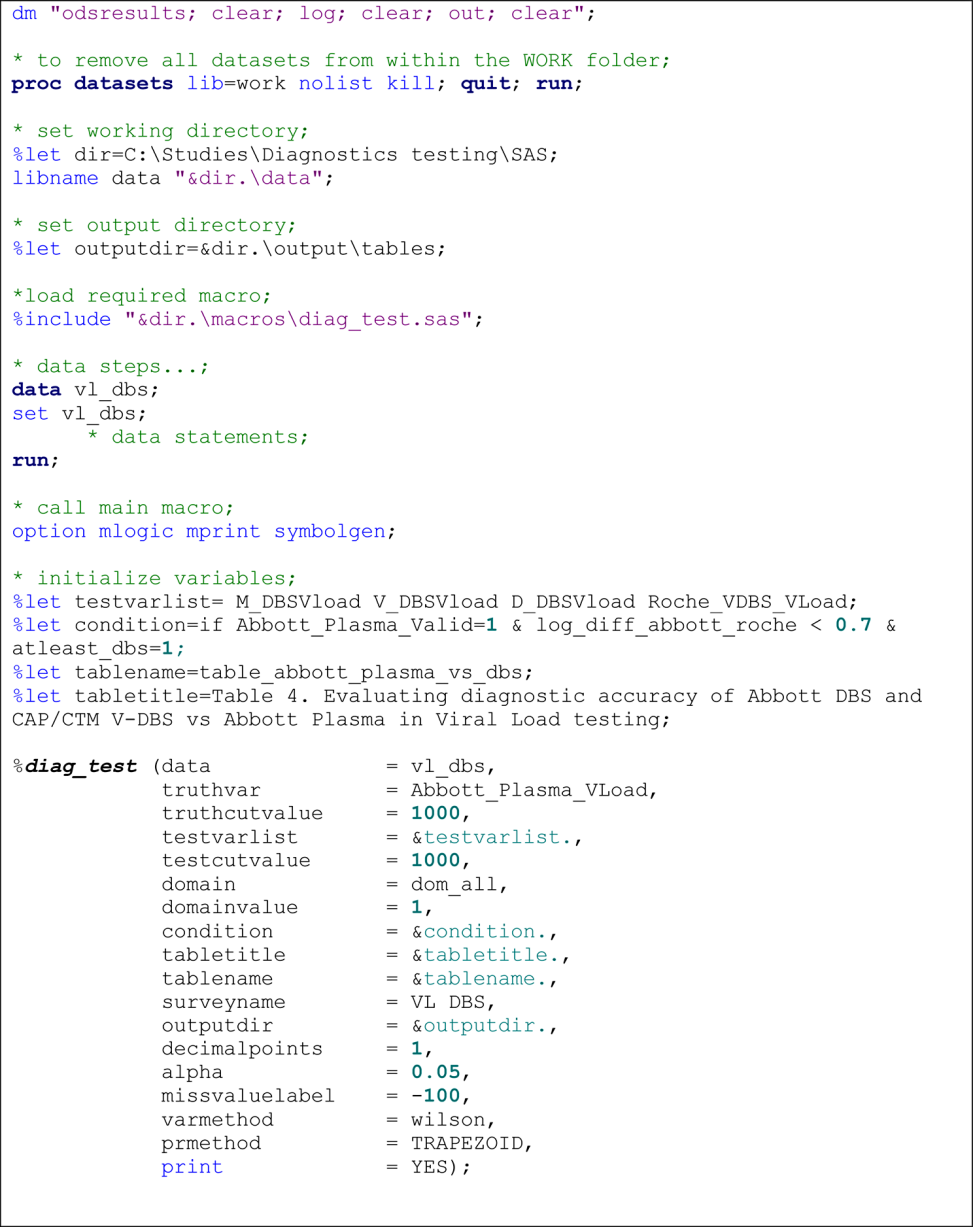
Sample *%diag_test* macro call

We used the macro to reproduce analysis results using data from published work on evaluation of dried blood spots (DBS) compared to plasma specimen as an alternative for HIV-1 viral load monitoring in resource-limited settings [50]. In brief, the World Health Organization (WHO) recommends viral load (VL) testing as the preferred method for diagnosing antiretroviral therapy treatment failure. Blood plasma specimen is the most preferred specimen type for VL for monitoring due to its lowest limit of detection compared to DBS specimen. Additionally, plasma VL testing reagents are costly and measured volume of blood must be prepared in a reference laboratory setting by a trained laboratory technician. DBSs on the other hand are easy to prepare in a resource-limited setting. For this study, DBSs were prepared from venous blood (V-DBS), microcapillary tubes (M-DBS) and directly spotting (D-DBS) and tested on the Abbott m2000 platform. Additionally, plasma and V-DBS specimens were also tested on the Roche COBAS Ampliprep/COBAS TaqMan (CAP/CTM) version 2.0 platform. Virologic failure (VF), defined as having ≥ 1000 copies/mL of virus, was used to classify the continuous viral load measure into two categories for both diagnostic and reference tests [50]. Figure 2 shows the macro call script used with this example.

Table 4 shows the publication-quality output after the macro was run. The output consists of seven [7] columns. Columns 1–2 display the variable label and value labels for cut-off threshold for the diagnostic test to be evaluated. Columns 3–5 display the variable label, value labels for cut-off threshold for the reference (gold standard) test and a 2 × 2 contingency table and confusion matrix summarizing the data. Columns 6–7 display the name of the diagnostic measure and corresponding point estimate and confidence interval. The output provides estimates for 16 measures of diagnostic testing for the user to select from and uses the Wilson score method to compute confidence intervals.

**Table 4:** Evaluating diagnostic accuracy of Abbott and Roche DBS vs. Abbott Plasma in VL testing

Diagnostic test(s)		Reference test (Abbott Plasma VL copies/mL)			Diagnostic accuracy measures (DAMs)	
Test category	Test result	>=1000	< 1000	Total	Measure	Estimate (95% CI)
M-DBS VL copies/ml	>=1000	213	26	239	Sensitivity (%)	90.3 (86.5–94.0)
					Specificity (%)	94.9 (92.9–96.8)
	< 1000	23	480	503	Positive Predictive Value (PPV) (%)	89.1 (85.2–93.1)
					Negative Predictive Value (NPV) (%)	95.4 (93.6–97.3)
	Total	236	506	742	Upward Misclassification (%)	5.1 (3.2–7.1)
					Downward Misclassification (%)	9.7 (6.0–13.5)
					False Omission Rate (FOR) (%)	4.6 (2.7–6.4)
					False Discovery Rate (FDR) (%)	10.9 (8.2–13.6)
					Positive Likelihood Ratio (LR+)	17.6 (12.1–25.6)
					Negative Likelihood Ratio (LR-)	0.1 (0.1–0.2)
					Diagnostic Accuracy (%)	93.4 (91.6–95.2)
					Disease Prevalence (%)	31.8 (28.5–35.2)
					Diagnostic Odds Ratio (DOR)	171.0 (95.4–306.5)
					Kappa Agreement	0.8 (0.8–0.9)
					Youden’s Index	0.9 (0.8–0.9)
					F-score	0.9 (0.9–0.9)
V-DBS VL copies/ml	>=1000	219	35	254	Sensitivity (%)	90.1 (86.4–93.9)
					Specificity (%)	93.1 (90.9–95.3)
	< 1000	24	475	499	Positive Predictive Value (PPV) (%)	86.2 (82.0–90.5)
					Negative Predictive Value (NPV) (%)	95.2 (93.3–97.1)
	Total	243	510	753	Upward Misclassification (%)	6.9 (4.7–9.1)
					Downward Misclassification (%)	9.9 (6.1–13.6)
					False Omission Rate (FOR) (%)	4.8 (2.9–6.7)
					False Discovery Rate (FDR) (%)	13.8 (10.8–16.8)
					Positive Likelihood Ratio (LR+)	13.1 (9.5–18.1)
					Negative Likelihood Ratio (LR-)	0.1 (0.1–0.2)
					Diagnostic Accuracy (%)	92.2 (90.2–94.1)
					Disease Prevalence (%)	32.3 (28.9–35.6)
					Diagnostic Odds Ratio (DOR)	123.8 (71.9–213.3)
					Kappa Agreement	0.8 (0.8–0.9)
					Youden’s Index	0.8 (0.8–0.9)
					F-score	0.9 (0.9–0.9)
D-DBS VL copies/ml	>=1000	208	28	236	Sensitivity (%)	88.1 (84.0–92.3)
					Specificity (%)	94.5 (92.5–96.5)
	< 1000	28	480	508	Positive Predictive Value (PPV) (%)	88.1 (84.0–92.3)
					Negative Predictive Value (NPV) (%)	94.5 (92.5–96.5)
	Total	236	508	744	Upward Misclassification (%)	5.5 (3.5–7.5)
					Downward Misclassification (%)	11.9 (7.7–16.0)
					False Omission Rate (FOR) (%)	5.5 (3.5–7.5)
					False Discovery Rate (FDR) (%)	11.9 (9.1–14.7)
					Positive Likelihood Ratio (LR+)	16.0 (11.1–23.0)
					Negative Likelihood Ratio (LR-)	0.1 (0.1–0.2)
					Diagnostic Accuracy (%)	92.5 (90.6–94.4)
					Disease Prevalence (%)	31.7 (28.4–35.1)
					Diagnostic Odds Ratio (DOR)	127.3 (73.6–220.4)
					Kappa Agreement	0.8 (0.8–0.9)
					Youden’s Index	0.8 (0.8–0.9)
					F-score	0.9 (0.9–0.9)
CAP/CTM V-DBS VL copies/ml	>=1000	219	343	562	Sensitivity (%)	94.4 (91.4–97.4)
					Specificity (%)	33.0 (28.9–37.1)
	< 1000	13	169	182	Positive Predictive Value (PPV) (%)	39.0 (34.9–43.0)
					Negative Predictive Value (NPV) (%)	92.9 (89.1–96.6)
	Total	232	512	744	Upward Misclassification (%)	67.0 (62.9–71.1)
					Downward Misclassification (%)	5.6 (2.6–8.6)
					False Omission Rate (FOR) (%)	7.1 (3.4–10.9)
					False Discovery Rate (FDR) (%)	61.0 (53.9–68.1)
					Positive Likelihood Ratio (LR+)	1.4 (1.3–1.5)
					Negative Likelihood Ratio (LR-)	0.2 (0.1–0.3)
					Diagnostic Accuracy (%)	52.2 (48.6–55.7)
					Disease Prevalence (%)	31.2 (27.9–34.5)
					Diagnostic Odds Ratio (DOR)	8.3 (4.6–15.0)
					Kappa Agreement	0.2 (0.2–0.2)
					Youden’s Index	0.3 (0.2–0.3)
					F-score	0.6 (0.5–0.6)

From Table 4, estimated prevalence of VF was about 32%. Overall, estimated DAMs indicate that all DBS tested on Abbott platform were highly comparable with plasma in VL testing. Specifically, sensitivity ranged from 88.1 to 90.3%, whereas specificity ranged from 93.1 to 94.9%. Upward and downward misclassification were also small ranging from 5.1 to 6.9% and 9.7–11.9% respectively. Predictive values were also high and ranged from 86.2 to 89.1% for PPV and from 94.5 to 95.4% for NPV. This means that the DBS can discriminate and/or predict with great precision clients with VF and those without VF. Likelihood ratio estimates show strong evidence of diagnostic accuracy with values for LR + ranging from 13.1 to 17.6. Values for LR- were close to zero with an estimate of about 0.1 for all DBS. Diagnostic accuracy ranged from 92.2 to 93.4% whereas DOR estimates ranged from 123.8 to 171.0. Values for Kappa statistic, Youden’s index and F-score values ranged from 0.8 to 0.9 for all DBS sample types.

V-DBS tested on the Roche CAP/CTM platform performed poorly when compared to plasma VL. Sensitivity, downward misclassification and NPV were optimal at 94.4%, 5.6%, and 92.9% respectively. However, specificity, upward misclassification, PPV were sub-optimal at 33.0%, 67.0% and 39.0% respectively. There was also weak evidence of diagnostic accuracy based on sub-optimal values for Diagnostic accuracy (52.2%), LR+ (1.4), DOR (8.3), Kappa statistic (0.2), Youden’s index (0.3) and F-score (0.6).

**Fig. 3:**
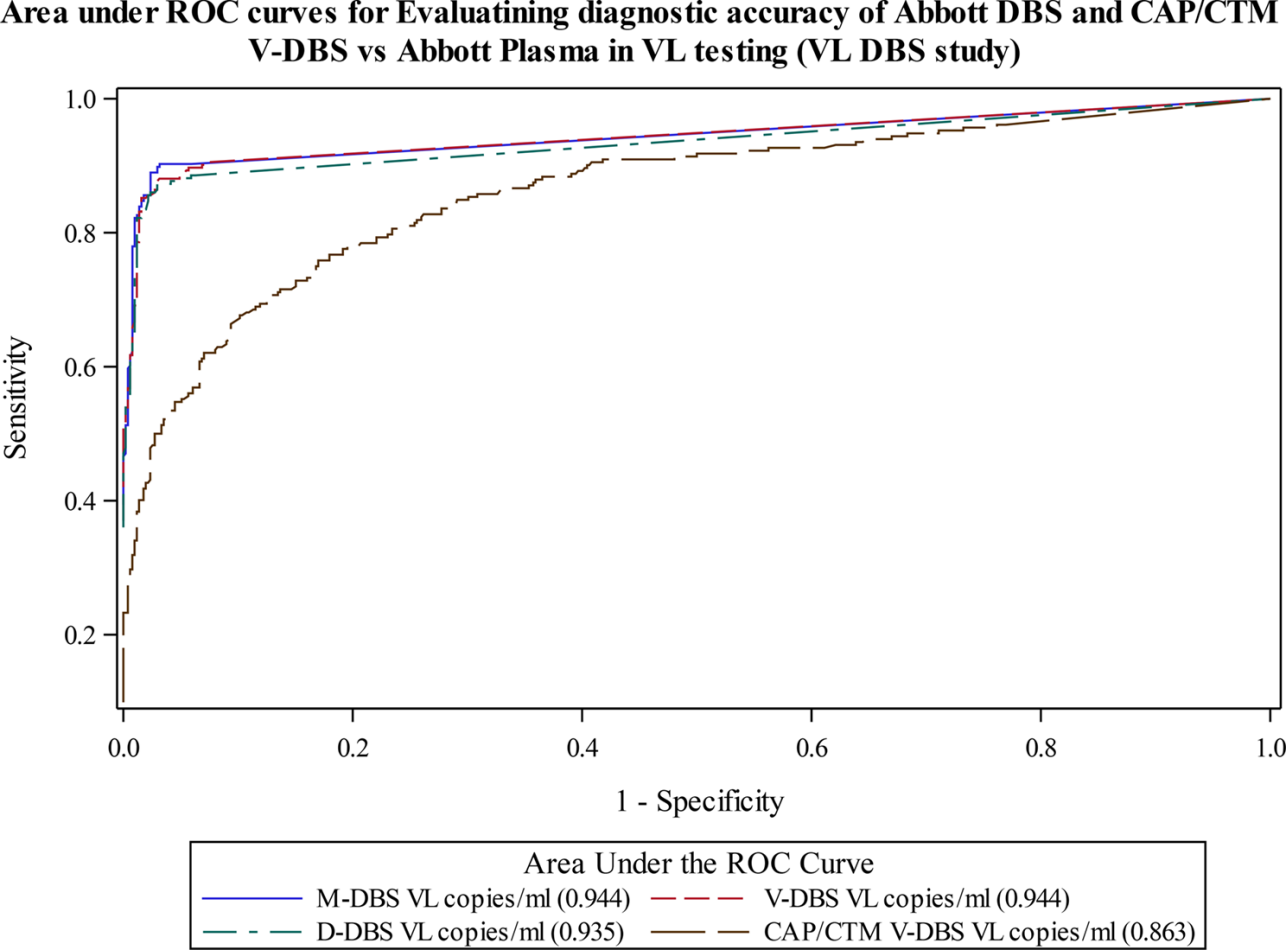
AUROCs evaluating diagnostic accuracy of Abbott and Roche DBS vs. Abbott Plasma in VL testing

**Fig. 4:**
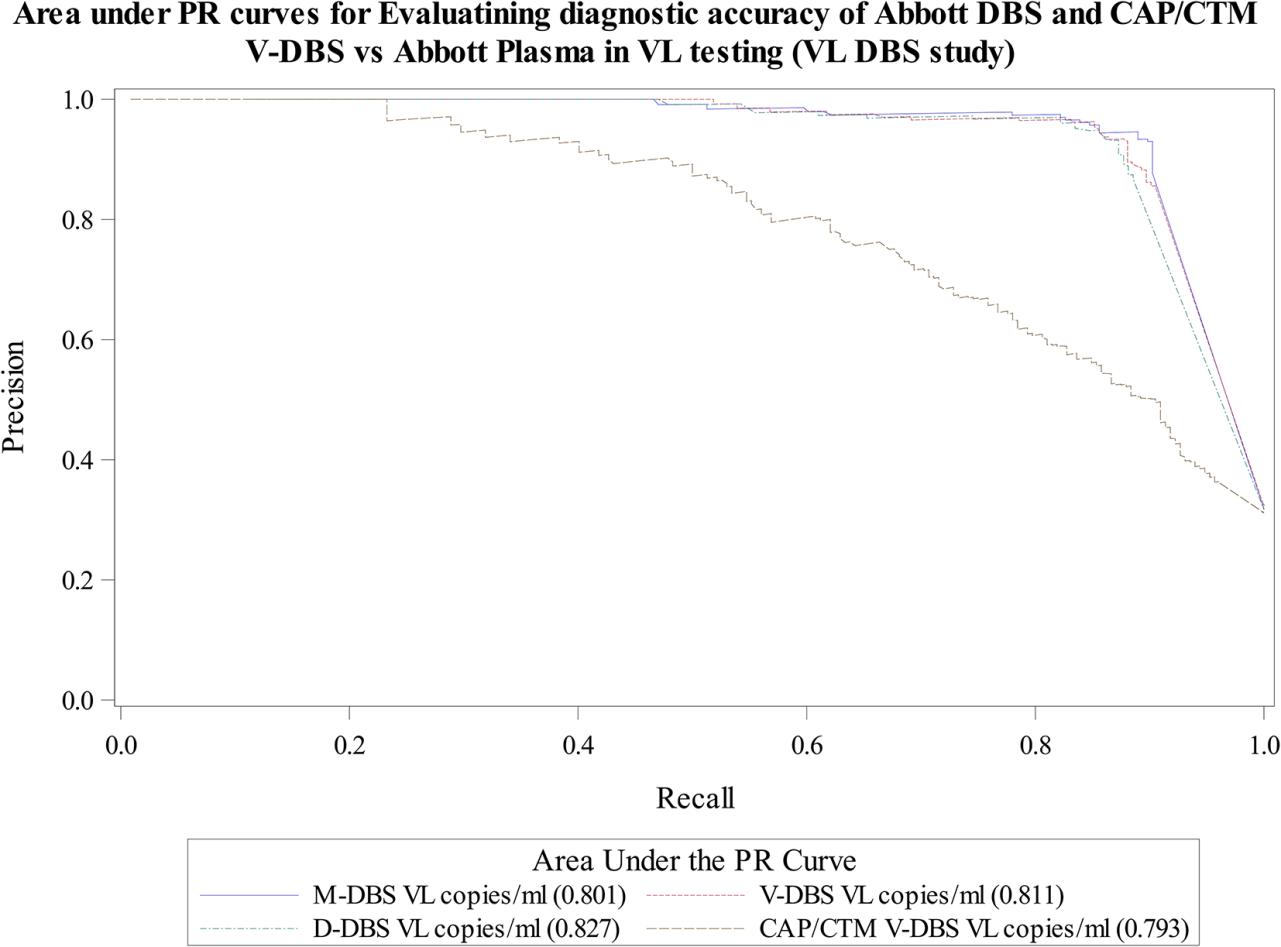
AUPRCs evaluating diagnostic accuracy of Abbott and Roche DBS vs. Abbott Plasma in VL testing

Figure 3 shows ROC with corresponding AUC overlaid in one graph and as displayed. AUROC for DBS tested on Abbott platform ranged from 0.935 to 0.944 compared to a lower AUROC of 0.863 for V-DBS tested on the Roche CAP/CTM platform. The AUPRC results shown in Fig. 4 are also similar and complement findings shown in Table 4. In summary, findings from this and other similar studies showed that DBS tested on Abbott platform could be used as an alternative to Plasma blood in VL monitoring of HIV positive clients in resource limited setting as it provided the desirable reasonable sensitivity and specificity (> 85%) [51].

### Example: application of *%diag_test* macro to evaluate performance of multiple classification machine learning algorithms

**Fig. 5:**
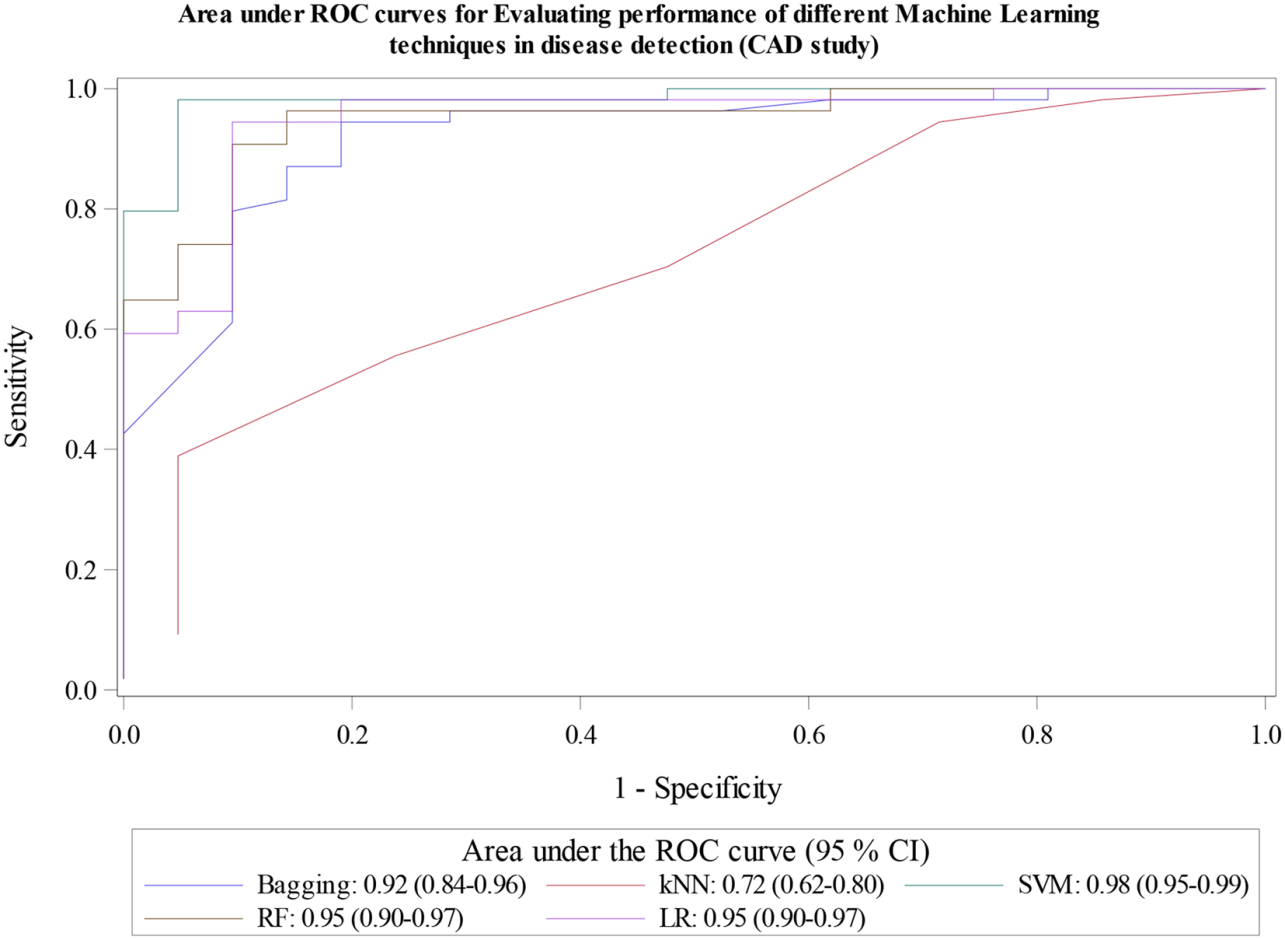
AUROCs Evaluating performance of different Machine Learning techniques in disease detection (CAD study)

**Fig. 6:**
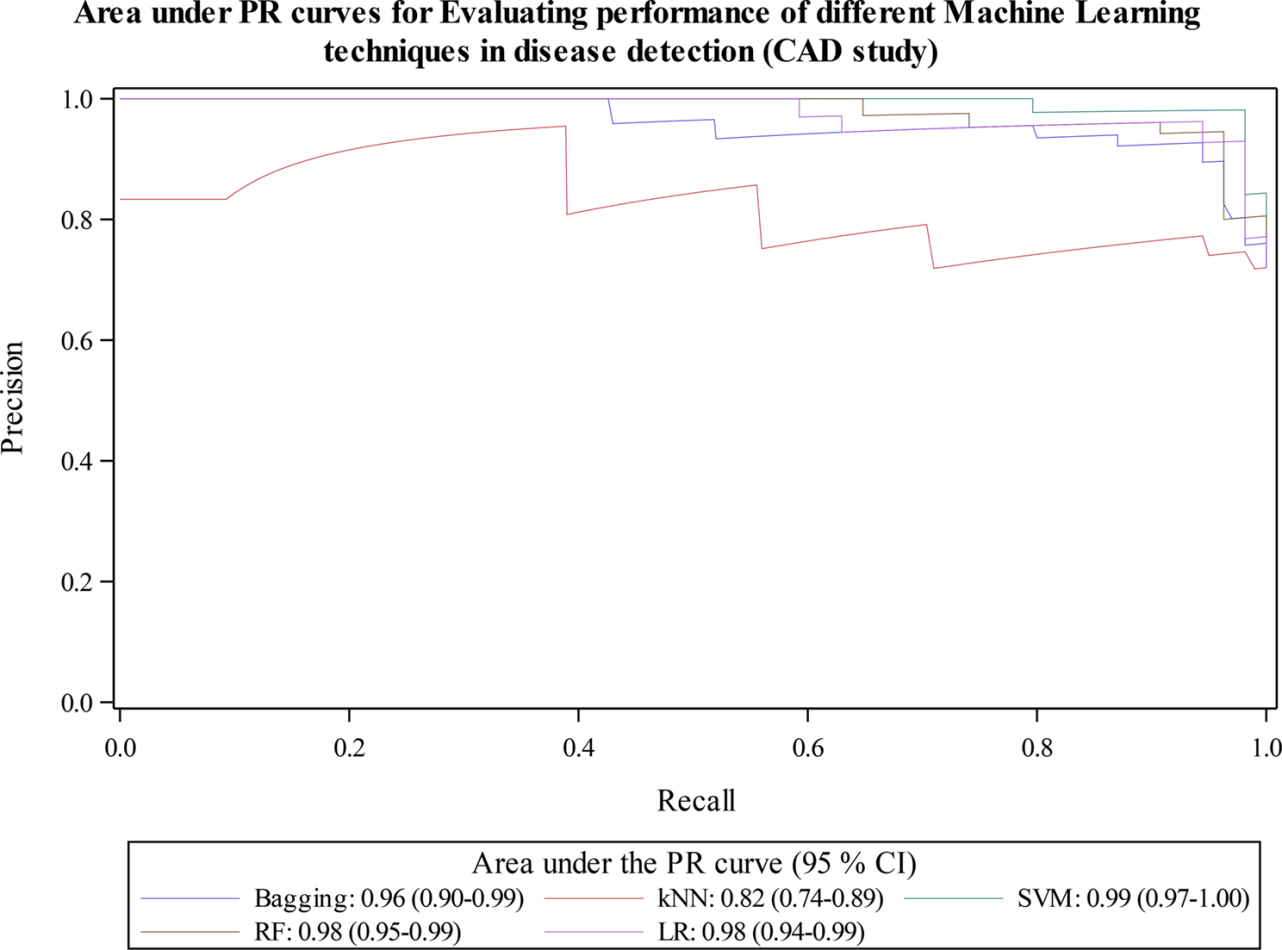
AUPRCs Evaluating performance of different Machine Learning techniques in disease detection (CAD study)

We also applied the macro to evaluate performance of multiple classification machine learning algorithms based on published work on coronary artery disease (CAD) detection [52]. In summary, CAD is a common type of cardiovascular disease (CVD) which fatal if not treated. The authors trained the models on 75% of the data (227 observations) and evaluated performance of five binary classification machine learning algorithms in detection of CAD using the renaming 25% (76 observations). The algorithms were: Logistic Regression (LR), Classification Tree with Bagging (Bagging CART), Random Forest (RF), Support Vector Machine (SVM), and K-Nearest Neighbors (KNN). Since we did not have the predicted probabilities, we recreated them by rerunning the models based on author specifications. We then used the SAS macro to compare their performance in predicting detecting CAD. While the authors of the original study evaluated models based on only Sensitivity, Specificity, Accuracy and AUC measures, we present all the diagnostic measures. The dataset is publicly available and is described in detail here [52, 53]. Outputs from the macro are like the ones from the previous example and are briefly discussed below in Table 5; Figs. 5 and 6.

**Table 5:** Evaluating performance of different machine learning techniques in disease detection (CAD study)

Diagnostic test(s)		Reference test (CAD)			Diagnostic accuracy measures (DAMs)	
Test category	Test result	>=0.5	< 0.5	Total	Measure	Estimate (95% CI)
Bagging	>=0.5	50	4	54	Sensitivity (%)	92.6 (82.4–97.1)
					Specificity (%)	81.0 (60.0–92.3)
	< 0.5	4	17	21	Positive Predictive Value (PPV) (%)	92.6 (82.4–97.1)
					Negative Predictive Value (NPV) (%)	81.0 (60.0–92.3)
	Total	54	21	75	Upward Misclassification (%)	19.0 (7.7–40.0)
					Downward Misclassification (%)	7.4 (2.9–17.6)
					False Omission Rate (FOR) (%)	19.0 (7.7–40.0)
					False Discovery Rate (FDR) (%)	7.4 (2.9–17.6)
					Positive Likelihood Ratio (LR+)	4.9 (2.0–11.8)
					Negative Likelihood Ratio (LR-)	0.1 (0.0–0.2)
					Diagnostic Accuracy (%)	89.3 (80.3–94.5)
					Disease Prevalence (%)	72.0 (61.0–80.9)
					Diagnostic Odds Ratio (DOR)	53.1 (12.0–236.0)
					Kappa Agreement	0.7 (0.6–0.9)
					Youden’s Index	0.7 (0.6–0.8)
					F-score	0.9 (0.8–1.0)
kNN	>=0.5	51	15	66	Sensitivity (%)	94.4 (84.9–98.1)
					Specificity (%)	28.6 (13.8–50.0)
	< 0.5	3	6	9	Positive Predictive Value (PPV) (%)	77.3 (65.8–85.7)
					Negative Predictive Value (NPV) (%)	66.7 (35.4–87.9)
	Total	54	21	75	Upward Misclassification (%)	71.4 (50.0–86.2)
					Downward Misclassification (%)	5.6 (1.9–15.1)
					False Omission Rate (FOR) (%)	33.3 (12.1–64.6)
					False Discovery Rate (FDR) (%)	22.7 (14.3–34.2)
					Positive Likelihood Ratio (LR+)	1.3 (1.0–1.7)
					Negative Likelihood Ratio (LR-)	0.2 (0.1–0.7)
					Diagnostic Accuracy (%)	76.0 (65.2–84.2)
					Disease Prevalence (%)	72.0 (61.0–80.9)
					Diagnostic Odds Ratio (DOR)	6.8 (1.5–30.5)
					Kappa Agreement	0.3 (0.0–0.5)
					Youden’s Index	0.2 (0.1–0.3)
					F-score	0.9 (0.8–0.9)
SVM	>=0.5	52	1	53	Sensitivity (%)	96.3 (87.5–99.0)
					Specificity (%)	95.2 (77.3–99.2)
	< 0.5	2	20	22	Positive Predictive Value (PPV) (%)	98.1 (90.1–99.7)
					Negative Predictive Value (NPV) (%)	90.9 (72.2–97.5)
	Total	54	21	75	Upward Misclassification (%)	4.8 (0.8–22.7)
					Downward Misclassification (%)	3.7 (1.0–12.5)
					False Omission Rate (FOR) (%)	9.1 (2.5–27.8)
					False Discovery Rate (FDR) (%)	1.9 (0.3–9.9)
					Positive Likelihood Ratio (LR+)	20.2 (3.0–137.0)
					Negative Likelihood Ratio (LR-)	0.0 (0.0–0.2)
					Diagnostic Accuracy (%)	96.0 (88.9–98.6)
					Disease Prevalence (%)	72.0 (61.0–80.9)
					Diagnostic Odds Ratio (DOR)	520.0 (44.6–6058)
					Kappa Agreement	0.9 (0.8–1.0)
					Youden’s Index	0.9 (0.8–1.0)
					F-score	1.0 (0.9–1.0)
RF	>=0.5	52	4	56	Sensitivity (%)	96.3 (87.5–99.0)
					Specificity (%)	81.0 (60.0–92.3)
	< 0.5	2	17	19	Positive Predictive Value (PPV) (%)	92.9 (83.0–97.2)
					Negative Predictive Value (NPV) (%)	89.5 (68.6–97.1)
	Total	54	21	75	Upward Misclassification (%)	19.0 (7.7–40.0)
					Downward Misclassification (%)	3.7 (1.0–12.5)
					False Omission Rate (FOR) (%)	10.5 (2.9–31.4)
					False Discovery Rate (FDR) (%)	7.1 (2.8–17.0)
					Positive Likelihood Ratio (LR+)	5.1 (2.1–12.2)
					Negative Likelihood Ratio (LR-)	0.0 (0.0–0.2)
					Diagnostic Accuracy (%)	92.0 (83.6–96.3)
					Disease Prevalence (%)	72.0 (61.0–80.9)
					Diagnostic Odds Ratio (DOR)	110.5 (18.6–657.6)
					Kappa Agreement	0.8 (0.6–1.0)
					Youden’s Index	0.8 (0.7–0.9)
					F-score	0.9 (0.9–1.0)
LR	>=0.5	51	4	55	Sensitivity (%)	94.4 (84.9–98.1)
					Specificity (%)	81.0 (60.0–92.3)
	< 0.5	3	17	20	Positive Predictive Value (PPV) (%)	92.7 (82.7–97.1)
					Negative Predictive Value (NPV) (%)	85.0 (64.0–94.8)
	Total	54	21	75	Upward Misclassification (%)	19.0 (7.7–40.0)
					Downward Misclassification (%)	5.6 (1.9–15.1)
					False Omission Rate (FOR) (%)	15.0 (5.2–36.0)
					False Discovery Rate (FDR) (%)	7.3 (2.9–17.3)
					Positive Likelihood Ratio (LR+)	5.0 (2.0–12.0)
					Negative Likelihood Ratio (LR-)	0.1 (0.0–0.2)
					Diagnostic Accuracy (%)	90.7 (82.0–95.4)
					Disease Prevalence (%)	72.0 (61.0–80.9)
					Diagnostic Odds Ratio (DOR)	72.3 (14.7–355.9)
					Kappa Agreement	0.8 (0.6–0.9)
					Youden’s Index	0.8 (0.6–0.8)
					F-score	0.9 (0.9–1.0)

From Table 5, estimated prevalence of CAD was about 72.0%. Overall, estimated DAMs indicate that SVM model performed better with very high sensitivity of 96.3% and specificity of 95.2%. Upward and downward misclassification were also smallest at 4.8% and 3.7% respectively. Predictive values were also high at 98.1% for PPV and 90.9% for NPV. This means that the SVM model can detect/predict CAD with great precision. Likelihood ratio estimates show strong evidence of diagnostic accuracy with values for LR + and LR- at 20.2 and 0 respectively. Diagnostic accuracy was 96.0% whereas DOR was at 520. Values for Kappa statistic, Youden’s index and F-score were greater than or equal to 9.0.

Three models RF, LR and Bagging CART performed second best models. They had also high sensitivity which ranged from 92.6 to 96.3%. Specificity and Upward misclassification were the same for all models at 81.0% and 19% respectively. Downward misclassification was also small and ranged from 3.7 to 7.4%. Predictive values were also high and ranged from 92.6 to 92.9% for PPV and from 81.0 to 89.5% for NPV. Likelihood ratio estimates show evidence of diagnostic accuracy with values for LR + and LR- at 5% and 0% respectively. Diagnostic accuracy ranged from 89.3 to 92% whereas DOR estimates ranged from 53.1 to 110.5. Values for Kappa statistic, Youden’s index and F-score values ranged from 0.7 to 0.9 for all the three models.

kNN model performed poorly at detecting CAD. Sensitivity, downward misclassification and LR- were optimal at 94.4%, 5.6%, and 0.2 respectively. However, specificity and upward misclassification were sub-optimal at 28.6% and 71.4.0% respectively. There was also weak evidence of diagnostic accuracy based on sub-optimal values for Diagnostic accuracy (76.0%), LR+ (1.3), DOR (6.4), Kappa statistic (0.3) and Youden’s index (0.2). Figure 5 shows ROC with corresponding AUC. SVM model had highest AUC at 0.98 and kNN had the lowest at 0.72. RF, LR and Bagging CART models had AUC of 0.95, 0.95 and 0.92 respectively.

Figure 6 shows AUPRC results. SVM model had highest AUPRC at 0.99 and kNN had the lowest at 0.82. RF, LR and Bagging CART AUCPR values of 0.98, 0.98 and 0.96 respectively.

Results presented here provide evidence that SVM model was the best at detecting CAD which agrees with findings from original paper.

## Discussion

This paper describes a simple and generalizable SAS macro, %diag_test, for computing DAMs for the purpose of comparing diagnostic tests to a reference standard. The macro is automated to save on analysis time, reduce transcription (copy-pasting) errors, and produce publication-quality outputs in both Microsoft Word and Excel formats making it easy to transfer output to other platforms. It is flexible to allow users to specify desired significance level and precision of point estimates. Most available tools are only able to evaluate one diagnostic test at a time. The macro also uses subject-level data and creates a 2 × 2 contingency table and confusion matrix as part of output, and more than 15 different measures of evaluating diagnostic accuracy for the analyst to choose from. Additional diagnostic measures can easily be added by modifying the code. The macro further is easy to use as the analyst provides the data in a SAS format and specifies the “truth” and “test” variables names. It is generic to use in different settings such as the laboratory to evaluate performance of multiple diagnostic tests or in machine learning to compare performance of different classification algorithms.

### Limitations

While the macro serves as a potent instrument for analyzing data from diagnostic studies, it is subject to several limitations that users must consider. Firstly, as a generic tool, the macro does not offer interpretation of results; such interpretations are contingent upon factors like disease prevalence and spectrum. For example, in cases where a disease is highly infectious or carries significant complications, prioritizing sensitivity may be more critical than achieving high specificity. Conversely, when subsequent testing or treatments carry substantial risks or costs, a higher specificity might be favored over sensitivity [54]. Therefore, the user should liaise with a subject matter expert and qualified statistician for inference.

Another limitation pertains to the statistical methods used within the macro for estimating variances and confidence intervals of complex DAMs. The current version assumes a binomial distribution for all DAMs; however, this assumption may not hold for more intricate measures derived from confusion matrix elements—such as ratios or products of true positives, false positives, etc.—which do not follow a standard distribution and thus cannot be accurately modeled by a binomial distribution alone. This could lead to potential underestimation or overestimation of variance and confidence intervals for these complex DAMs. Users should be aware that alternative statistical approaches—such as bootstrapping, Bayesian methods, delta method, or simulation studies—may provide more accurate uncertainty quantification for these measures and should consider seeking additional expertise in these areas.

Furthermore, the macro does not perform any data cleaning tasks; it presumes that the dataset has been thoroughly cleansed prior to analysis. It expects variables to be correctly labeled and values converted into appropriate formats compatible with SAS before being fed into the macro call.

Lastly, the macro was designed specifically for use on Microsoft Windows platforms. Users operating on different systems will need to adjust parts of the code to align with their respective platform’s syntax structure. This may necessitate additional technical proficiency or support to ensure compatibility and functionality across diverse operating environments.

## Conclusion

In conclusion, we note that the macro is a powerful and more advanced analytic tool compared to existing ones as it provides a pool of measures to choose from and compare against to make concrete decisions regarding adoption or rejection of the diagnostic test. In addition, it builds on other existing tools developed by the authors to automate data analysis techniques [55, 56].

## Electronic supplementary material

Below is the link to the electronic supplementary material.

Supplementary Material 1

## Data Availability

The datasets used and/or analyzed during the current study are available from the corresponding author upon reasonable request. The source code for this SAS macro and data used for demonstration are available from the lead author and from GitHub repository at https://github.com/kmuthusi/diagnostic-testing-macro.

## Abbreviations

AIDS: Acquired Immune Deficiency Syndrome
AUC: Area Under Curve
CAP/CTM: COBAS Ampliprep/COBAS TaqMan
CDC: U.S. Centers for Disease Control and Prevention
DAMs: Diagnostic Accuracy Measures
DBS: Dried Blood Spots
D-DBS: Direct Spotting DBS
DOR: Diagnostic Odds Ratio
FDR: False Discovery Rate
FN: False Negative
FNR: False Negative Rate
FOR: False Omission Rate
FP: False Positive
FPR: False Positive Rate
HIV: Human Immunodeficiency Virus
LR-: Negative Likelihood Ratio
LR+: Positive Likelihood Ratio
M-DBS: Microcapillary DBS
NPV: Negative Predictive Value
ODS: Output Delivery System
PEFPAR: U.S. President’s Emergency Plan for AIDS Relief
PPV: Positive Predictive Value
PR: Precision-Recall
ROC: Receiver Operator Characteristic
TB: Tuberculosis
TN: True Negative
TNR: True Negative Rate
TP: True Positive
TPR: True Positive Rate
V-DBS: Venous DBS
VF: Virologic Failure
VL: Viral load
WHO: World Health Organization
